# Liver fat storage is controlled by HNF4α through induction of lipophagy and is reversed by a potent HNF4α agonist

**DOI:** 10.1038/s41419-021-03862-x

**Published:** 2021-06-11

**Authors:** Seung-Hee Lee, Vimal Veeriah, Fred Levine

**Affiliations:** SBP Medical Discovery Institute, La Jolla, CA USA

**Keywords:** Lipid signalling, Target validation

## Abstract

We report the discovery of strong HNF4α agonists and their use to uncover a previously unknown pathway by which HNF4α controls the level of fat storage in the liver. This involves the induction of lipophagy by dihydroceramides, the synthesis and secretion of which is controlled by genes induced by HNF4α. The HNF4α activators are N-trans caffeoyltyramine (NCT) and N-trans feruloyltyramine (NFT), which are structurally related to the known drugs alverine and benfluorex, which we previously showed to be weak HNF4α activators. In vitro, NCT and NFT induced fat clearance from palmitate-loaded cells. In DIO mice, NCT led to recovery of hepatic HNF4α expression and reduction of steatosis. Mechanistically, increased dihydroceramide production and action downstream of HNF4α occurred through increased expression of HNF4α downstream genes, including SPNS2 and CYP26A1. NCT was completely nontoxic at the highest dose administered and so is a strong candidate for an NAFLD therapeutic.

## Introduction

Fatty liver disease is a major cause of morbidity and mortality^1–3^. Excessive hepatic fat storage secondary to obesity causes hepatocyte dysfunction, termed non-alcoholic fatty liver disease (NAFLD). NAFLD progresses in many cases to non-alcoholic steatotic hepatitis (NASH), characterized by inflammation, fibrosis and hepatocyte death. In some individuals, this progresses further to cirrhosis and organ failure. Obesity-associated liver disease is a leading cause of liver transplantation^2^.

Fat is stored primarily in adipocytes and hepatocytes. Control of fat storage has been studied extensively in adipocytes^4^, where lipase activity is tightly regulated, including by insulin-mediated repression of hormone-sensitive lipase so that fat is stored in the fed state when insulin levels are high and released under starvation conditions when insulin is low. In contrast, the control of fat storage in the liver has been poorly understood.

Hepatic fat is an important source of fatty acids that can be mobilized to supply energy^5^. As in the adipocyte, hepatic fat is stored in lipid droplets, which are similar in most respects to thosein adipocytes^6^. However, in contrast to the adipocyte, the liver does not express hormone sensitive lipase and so a different but heretofore unknown mechanism must be used to control fat mobilization from hepatic lipid droplets. In particular, the mechanisms underlying the pathological fat storage that is the hallmark of NAFLD has not been clear. The mechanism by which fat storage in the liver is regulated must include two principal components: a mechanism to sense the amount of fat and a mechanism to control the amount of fat stored in hepatic lipid droplets. Because understanding the control of fat storage in the liver is critical to the development of therapeutics for fatty liver disease, there has been great interest in this area^6^.

Some time ago, we developed a cell based phenotypic screen for compounds that modulate the activity of a human insulin promoter-*GFP* transgene and used it to screen different compound libraries^7–11^. An important discovery is that our assay is highly sensitive to the level of activity of the nuclear hormone receptor hepatocyte nuclear factor 4α (HNF4α)^8^ and the T6PNE cells used for the assay have a low level of stored fat at baseline, unlike hepatoma cell lines, which we find to be highly steatotic, making it possible to more easily study the relationship between HNF4α activity and stored fat. HNF4α is expressed predominantly in the liver, intestine, pancreas, and kidney, where it plays important roles in metabolic homeostasis^12,13^. While the traditional view of HNF4a was that the ligand binding pocket (LBP) contained a tightly bound fatty acid that was not exchangeable, we and others have shown that HNF4a undergoes ligand exchange and that receptor activity can be regulated^7,8,14^.

Using the insulin promoter assay, we found that fatty acids inhibit HNF4α activity^8^, a previously undescribed activity, even though it was known that fatty acids bind in the HNF4α ligand binding pocket^15^. Also using the insulin promoter assay, we discovered both antagonists^8^ and agonists^7^ of HNF4α. The HNF4α activators were alverine and benfluorex, known drugs that have been used for irritable bowel syndrome and weight loss/type 2 diabetes, respectively. Of note, benfluorex has been studied in clinical trials for type 2 diabetes and proved to be effective at reducing HbA1c^16,17^. Unfortunately, both alverine and benfluorex were relatively weak activators, making it difficult to study the role of HNF4α in lipotoxic diseases. Thus, we decided to search for more potent activators.

To find more potent HNF4α activators, we examined compounds that had structural similarity with alverine or benfluorex. The plant-based compounds N-trans caffeoyltyramine (NCT), and N-trans feruloyltyramine (NFT) which are associated with plant cell walls as part of a damage response^18^, were found to be more potent activators of the human insulin promoter-GFP transgene in T6PNE cells. Of note, NFT is derived from NCT by the action of caffeoyltyramine-O-methyltransferase^19^. NCT and NFT induced clearance of fat from the T6PNE cells used in the insulin promoter assay and NCT was studied in vivo, where it reversed hepatic steatosis. The mechanism by which HNF4α affects hepatic fat storage is induction of lipophagy, a form of autophagy that involves fusion of lipid droplets with lysosomes and lipid hydrolysis through lysosomal acid lipase^20^. This is a mechanism distinct from that regulating adipocyte fat storage through hormone-sensitive lipase.

The data presented here demonstrate that HNF4α meets both of the principal requirements for a molecule that can mediate the control of hepatic lipid storage. It senses fat directly by fatty acid binding to the HNF4α ligand binding pocket (LBP), which controls HNF4α activity. The level of HNF4α activity then determines the extent of lipophagy, which releases fat from lipid vesicles in hepatocytes, thus regulating the amount of fat stored in the liver (Supplementary Fig. 1).

## Results

### Discovery of HNF4α activators

**Fig. 1:**
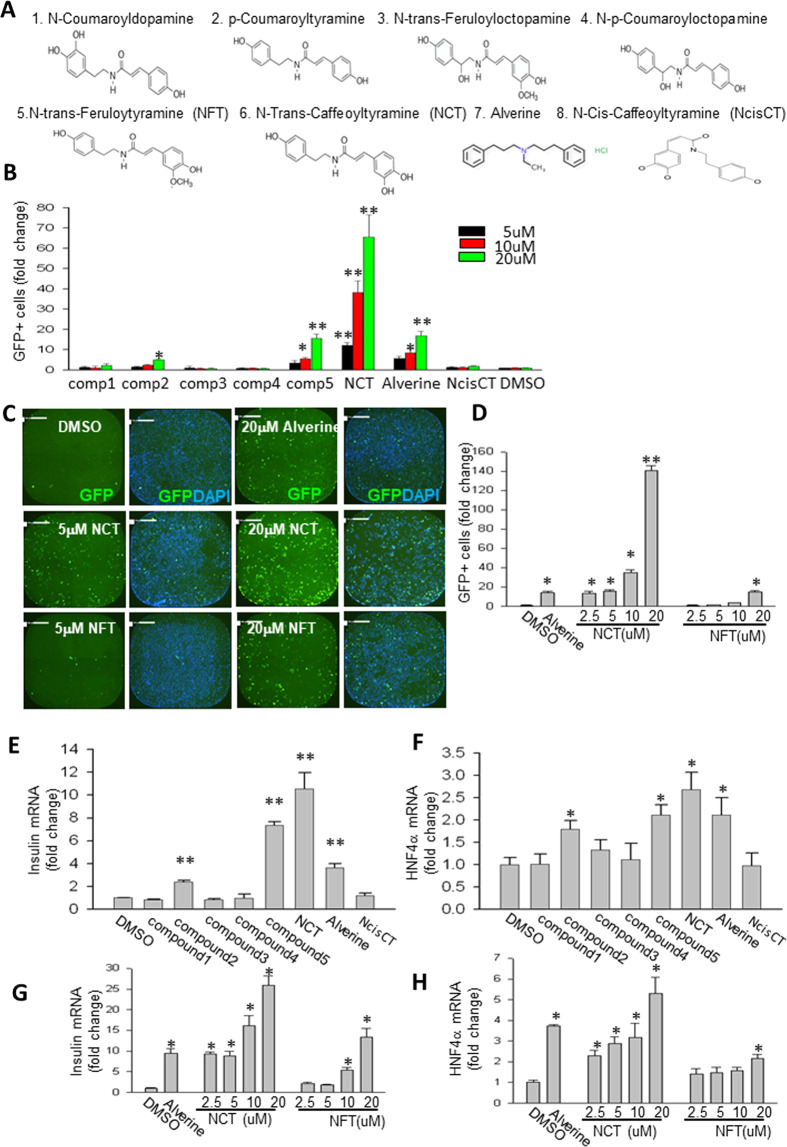
Discovery of potent HNF4α agonists. **A** Structures of tested compounds. **B** Assay for HNF4a activity. Compounds from **A** were assayed as previously described^10^. Briefly, T6PNE cells treated with tamoxifen (0.5 μM) to induce the activity of the E47^MER^ transgene^10^, were treated with the indicated compounds at 3 concentrations (5 μM, black, 10 μM, red, 20 μM, green) for 3 days, followed by fixation, staining with DAPI for nuclear visualization, and imaging for quantification of the percentage of cells expressing the insulin promoter-GFP transgene in the T6PNE cells (*N* = 6) using a Celigo imaging cytometer. **C** Representative images of wells containing the DMSO negative control, alverine positive control, and the positive compounds NCT and NFT (green is from insulin promotor-GFP expression and blue is DAPI nuclear staining, scale bar = 500 μm). **D** Quantification of GFP-positive cells, reflecting activity of the human insulin promoter-*GFP* transgene in T6PNE cells, was done with multiple doses of NCT and NFT to demonstrate dose-responsiveness using a Celigo imaging cytometer (*N* = 14). **E**, **F** *Insulin* and *HNF4α* mRNA levels on compounds from A were measured by qPCR as an additional measure of HNF4α activity (compound concentration was 20 μM, *N* = 4). **G**, **H** *Insulin* and *HNF4α* mRNA levels were measured by qPCR with multiple doses of NCT and NFT (*N* = 4). Values represent the mean ± SE. *p < 0.05, **p < 0.01 (vs DMSO).

Previously, we screened a library of known drugs and discovered that alverine and benfluorex, which are structurally similar but used for completely distinct indications, were activators of HNF4α^7^. Because alverine and benfluorex were fairly weak HNF4α activators, finding stronger activators was compelling. Thus, we tested some compounds that had structural similarity with alverine and benfluorex to find additional HNF4α activators (Fig. 1A). N-trans caffeoyltyramine (NCT) and N-trans-feruloyltyramine (NFT) were reproducibly positive hits (Fig. 1B–D). NCT was the strongest inducer of insulin promoter activity, while NFT was approximately as active as alverine. As expected for nuclear receptor ligands, which are well-known for having highly sensitive structure-activity relationships^21^, small structural differences resulted in large changes in activity (Fig. 1B). NCT, which was more potent than NFT, differs from NFT by a single methyl group. In plants, NCT is converted to NFT by caffeoyltyramine-O-methyltransferase^19^, resulting in generally higher levels of NFT than NCT^18^. There was no estrogenic or PPARγ receptor agonist activity, both of which can produce false positives in the assay^8,10^ (Supplementary Fig. 2A, B)

Both NCT and NFT, and to a lesser degree N-p-coumaroyltyramine, increased *INS* and *HNF4α* mRNA (Fig. 1E, F). The increase in *HNF4α* mRNA is particularly important, since *HNF4α* gene expression is the best indicator of HNF4α activity in our hands, as the protein acts on its own promoter through a positive feedback loop^7,8,22,23^. Both NCT and NFT exhibited dose-responsiveness in the *INS* promoter assay (Fig. 1D) and with respect to their ability to increase *INS* and *HNF4α* mRNA levels (Fig. 1G, H). We believe that it is likely that fatty acids and the HNF4a agonists compete for occupancy of the HNF4a LBP and that this could account for threshold effects seen in the dose-response curves.

### NCT and NFT act directly on HNF4α

**Fig. 2:**
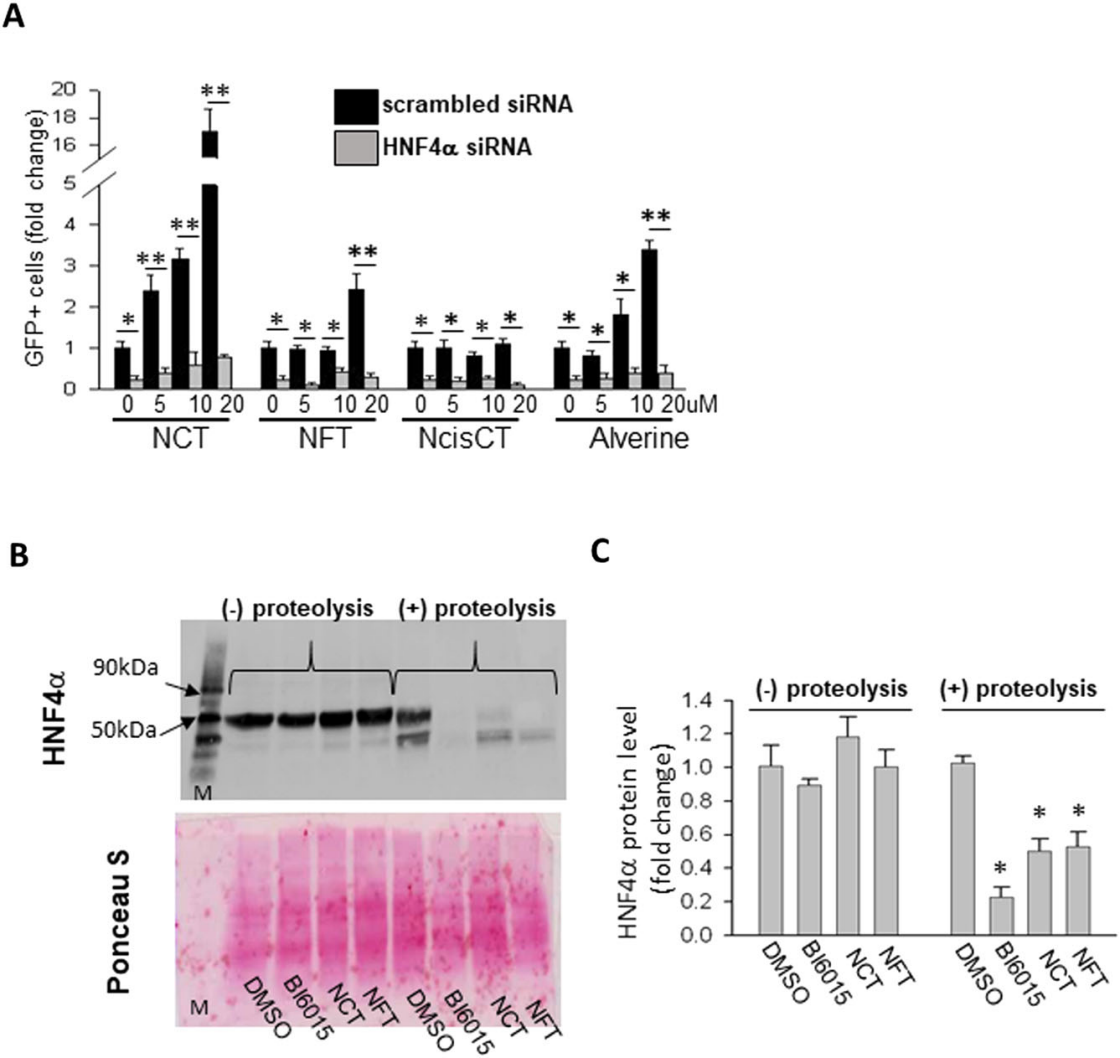
NCT and NFT are HNF4α agonists. **A** HNF4α siRNA blocked the effect of HNF4α agonists. Scrambled or HNF4α siRNAs were transfected into T6PNE cells 2 days before compound administration. Compounds at the indicated concentrations were treated for an additional 2 days. The cells were analyzed for the percentage of cells expressing the insulin promoter-*GFP* transgene (*N* = 6). **B** DARTS assay to detect effect of compounds on HNF4α protease sensitivity. For the DARTS assay, HepG2 cells were treated with DMSO (lane 1), BI6015 (lane 2), NCT (lane 3), or NFT (lane 4) at a concentration of 40 or 80 μM for 16 h. Total cell protein was extracted and each sample was split into two aliquots for proteolysis without (−) or with (+) subtilisin and analyzed by Western blotting for HNF4α as done previously^7^. After detection of HNF4α, the membrane was stained with Ponceau S (magenta color) as a control to ensure that the compounds did not induce nonspecific proteolysis (Lane M has MW markers). All compounds were run on the same gel. **C** The HNF4α level was quantified by ImageJ using the Western blots from panel **B**. Values represent the mean ± SE of 3 biological replicates, *p < 0.05, **p < 0.01 (vs scrambled siRNA or DMSO).

A prediction if the compounds act on HNF4α is that *HNF4α* siRNA should ablate their effect. Consistent with that prediction, *HNF4α* siRNA repressed the effect of NCT and NFT on the *INS* promoter (Fig. 2A, representative images in Supplementary Fig. 3).

Binding of a compound to its target is expected to alter the structure of the target protein. This can often be detected as a change in the sensitivity to proteolytic cleavage, which is the basis for the DARTS assay^24^. We used this previously to determine whether compounds induce a conformational change in HNF4α, thus demonstrating a direct effect on the protein^7,8^. Consistent with our previous results, the potent HNF4α antagonist BI6015 induced a conformational change in HNF4α (Fig. 2B, C). NCT and NFT also induced a change in HNF4α proteolytic sensitivity, as expected if they act directly (Fig. 2B, C).

### NCT induces fat clearance from cells

**Fig. 3:**
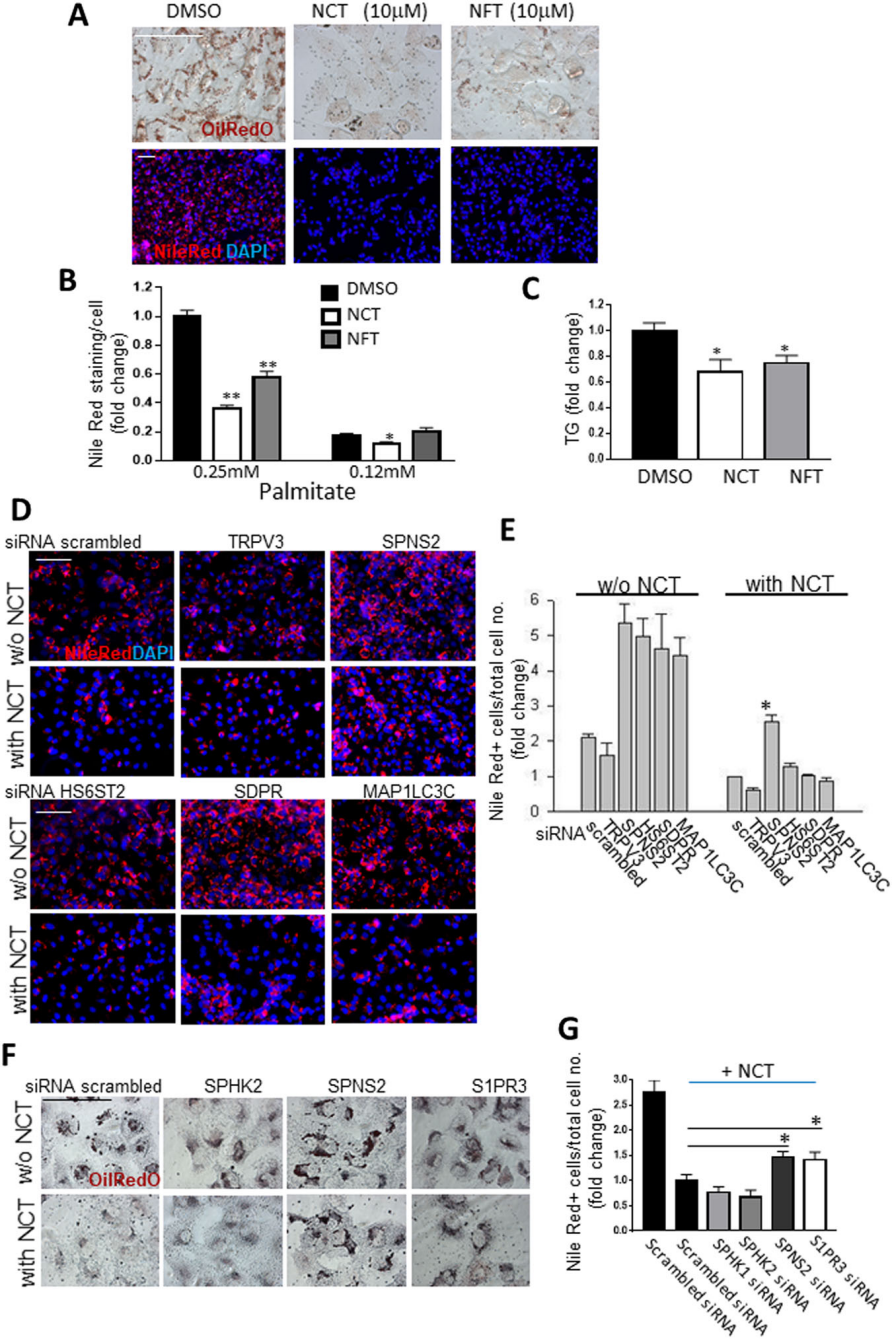
NCT and NFT induce fat clearance and act through SPNS2. For **A**–**C** T6PNE cells were treated with 0.25 mM palmitate and 10μM NCT or NFT for 2 days. **A** Photomicrographs of representative wells stained for fat with Oil Red O (upper panels) or Nile Red (lower panels). **B** Quantification of Nile Red staining was done on a per cell basis using a Celigo imaging cytometer (*N* = 14). **C** Triglyceride (TG) level was normalized to cellular protein measured by BCA and fold change was calculated relative to DMSO control (*N* = 6). **D** Candidate genes induced by NCT that have a role in fat metabolism were screened for a role in NCT-induced fat clearance using siRNAs. T6PNE cells were transfected with scrambled or target siRNAs. Two days later, DMS O or NCT plus palmitate (0.25 mM) was added for 2 days, followed by harvesting or analysis of the level of fat by Nile Red. Shown are representative photomicrographs. *N* = 4 biological replicates. **E** Quantification of Nile Red-positive cells processed as in **D**. The percent of total cells with Nile Red staining greater than a predetermined threshold was determined for each siRNA and normalized to the scrambled siRNA for that gene to calculate the fold change using a Celigo imaging cytometer. *N* = 4 biological replicates. **F** SPNS2 and S1PR3, but not SPHK2, are required for NCT-induced fat clearance. Cells were treated with siRNAs to the indicated genes plus NCT and palmitate as in panel **A**, followed by staining with Oil Red O. Shown are representative photomicrographs. **G** Quantification of cellular fat detected by Nile Red staining was done as in panel **E**. *N* = 4 biological replicates. Values represent the mean ± SE. *p < 0.05, **p < 0.01 (vs DMSO for **A**–**C** or scrambled siRNA for **D**–**G**). Scale bar = 100 μm.

The HNF4α antagonist BI6015 caused hepatic steatosis in vitro and in vivo^8^, and genetic deletion of *HNF4α* leads to hepatic steatosis^25^. Alverine and benfluorex were ascertained in a modification of the insulin promoter assay in which the level of insulin promoter activity was repressed with palmitate. In that modified assay, alverine and benfluorex reversed the fatty acid-mediated repression of the human insulin promoter^7^. Thus, it was logical to ask whether more potent HNF4α agonists could alleviate steatosis. Cells were treated with 0.25 mM palmitate for 2 days in the presence and absence of NCT or NFT (10 μM). As demonstrated by Oil Red O and Nile Red staining, cells treated with NCT or NFT had less stored fat than control cells (Fig. 3A, quantified in B, Supplementary Fig. 2C). This was further demonstrated by quantification of the cellular triglyceride (TG) level (Fig. 3C), which produced the same result as the Nile Red staining, validating the accuracy of that assay.

### The sphingosine-1-phosphate transporter SPNS2 is required for fat clearance by NCT but S1P does not play a role

Given that HNF4α is a transcription factor, we believed that the mechanism by which the newly discovered HNF4α agonists caused reversal of cellular steatosis would involve genes downstream of HNF4α. Genes with mRNA levels that were affected by both NCT and NFT and that were involved in lipid metabolism were tested for a role in fat clearance downstream of HNF4α by inhibiting their expression using siRNA (siRNA validation in Supplementary Fig. 4). Of the genes tested, siRNA to only one, *SPNS2*, blocked the effect of NCT on fat clearance (Fig. 3D, quantified in E). *SPNS2* encodes a transporter for sphingosine-1-phosphate (S1P) that moves it from the intracellular to the extracellular space. Of note, palmitate, which was used to induce steatosis and which is the major fat consumed by humans^26^ is a precursor for S1P synthesis^27^. An S1P analog that causes immunosuppression, (FTY720, aka fingolimod), has been approved for the treatment of multiple sclerosis^28^. S1P is synthesized from sphingosine by the action of sphingosine kinases (*Sphk1, 2*)^29^. T6PNE cells express only *Sphk2* to a significant degree (GEO Accession GSE18821, GSE33432) and siRNA to Sphk2 had no effect on fat clearance induced by NCT (Fig. 3F, quantified in G) eliminating S1P as the molecule responsible for inducing fat clearance.

Once transported outside cells by SPNS2, S1P binds to a receptor in the GPCR family of signaling receptors, of which there are five family members (*S1PR1-5*). S1PR signaling plays an important role in diverse cell processes, but particularly in immune responses^30^. Only one member of the S1PR family, *S1PR3*, is expressed in T6PNE cells (GEO Accession GSE18821, GSE33432). *S1PR3* siRNA blocked the ability of NCT to induce fat clearance (Fig. 3F, quantified in G), consistent with a model in which a molecule transported by SPNS2 that then stimulates S1PR signaling triggers fat clearance. However, this begged the question of the identity of that molecule.

### Dihydroceramides are required for fat clearance by NCT

**Fig. 4:**
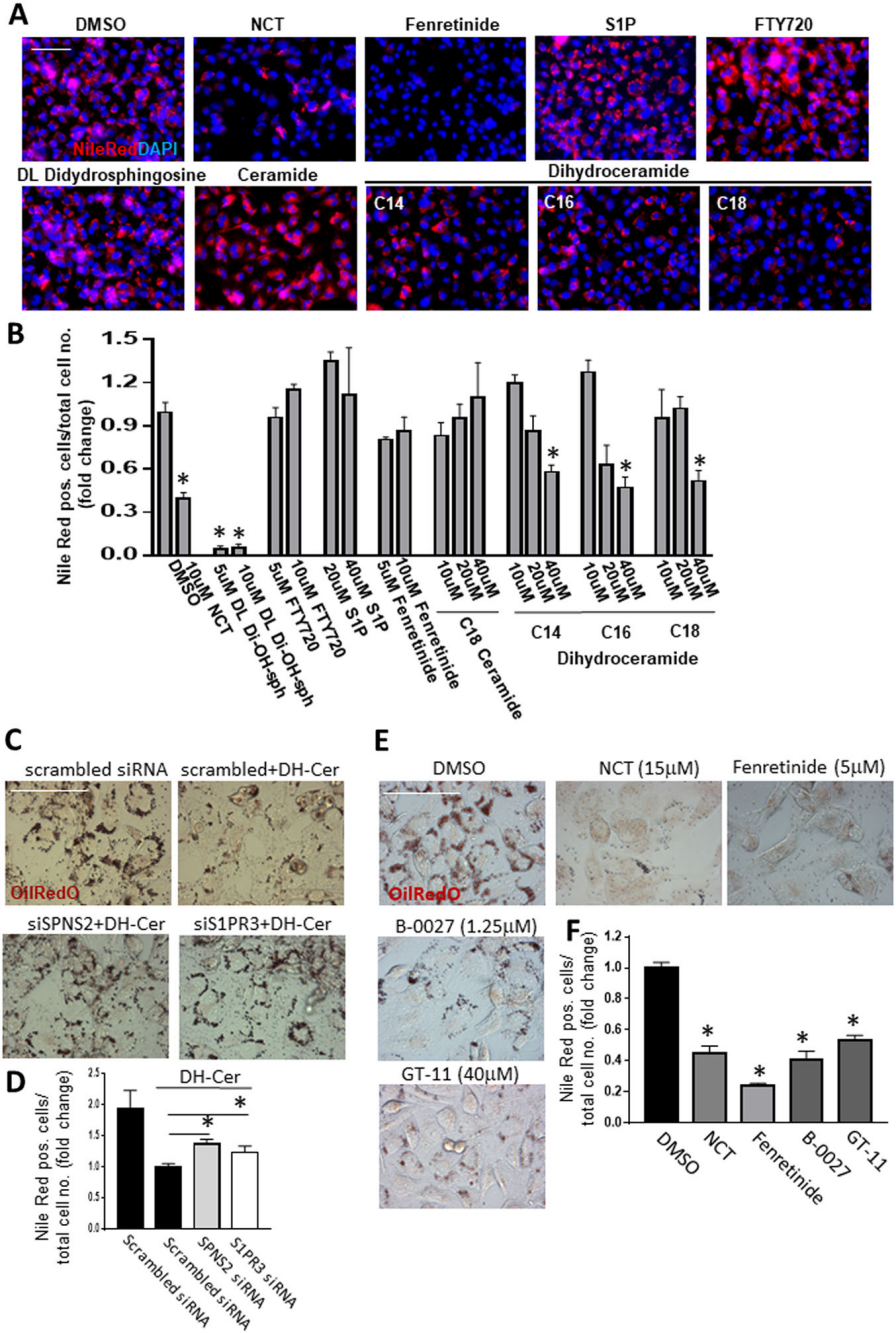
Dihydroceramides (DH-Cer) induce fat clearance. **A** Representative photomicrographs of T6PNE cells treated for 2 days with 0.25 mM palmitate and the indicated compounds, followed by staining for fat with Nile Red. **B** Quantification of Nile Red staining from **A**. **C** Inhibition by siRNA to SPNS2 or S1PR3 of fat clearance induced by DH-Cer. T6PNE cells were transfected with siRNAs to SPNS2 or S1PR3. Two days later, DH-Cer was added for 2 days, followed by staining with Oil Red O. **D** Quantification of the number of cells positive for Nile red from **C**, demonstrating that DH-Cer-induced fat clearance requires SPNS2 and S1PR3. **E** DES-1 inhibitors GT-11and B-0027 increase fat clearance. T6PNE cells were treated with 0.25 mM palmitate and DMSO or NCT. **F** Quantification of the Nile Red cells shown in **E**. Values represent the mean ± SE of 6 biological replicates, *p < 0.05 (vs DMSO or scrambled siRNA). Scale bar = 100 μm.

To our surprise, neither S1P nor FTY720 had any effect on fat clearance (Fig. 4A, quantified in B). However, because of the known roles of SPNS2 and S1PR3, molecules structurally related to S1P and that could be acted on by SPNS2 and S1PR3 were the obvious candidates for being the effectors in fat clearance induced by NCT. De novo S1P biosynthesis begins with palmitate and serine and proceeds through dihydrosphingosine, dihydroceramide, ceramide, and sphingosine^27^ (Supplementary Fig. 1). Dihydrosphingosines have been shown to be transported by SPNS2^31^, but had no effect on fat clearance (Fig. 4A, B). In contrast, multiple dihydroceramides were highly effective at inducing fat clearance (Fig. 4A, B). This suggests that, like S1P and dihydrosphingosines, dihydroceramides are transported by SPNS2 and act through S1PRs to effect fat clearance from cells (Fig. 4C, quantified in D).

If dihydroceramides are the active molecule in fat clearance, inhibition of their conversion to ceramides by dihydroceramide desaturase 1 (DES1) should increases their level and promote fat clearance (Supplementary Fig. 1). Fenretinide is a synthetic retinoid derivative that inhibits DES1^32^ but it has multiple other targets as well^33^. It was strongly positive in the fat clearance assay (Fig. 4A, B), as were the more specific DES1 inhibitors GT-11 and B-0027 (Fig. 4E, quantified in F) This provides further evidence that dihydroceramides are the active species responsible for the ability of NCT to induce fat clearance from cells.

### NCT promotes fat clearance by inducing lipophagy

**Fig. 5:**
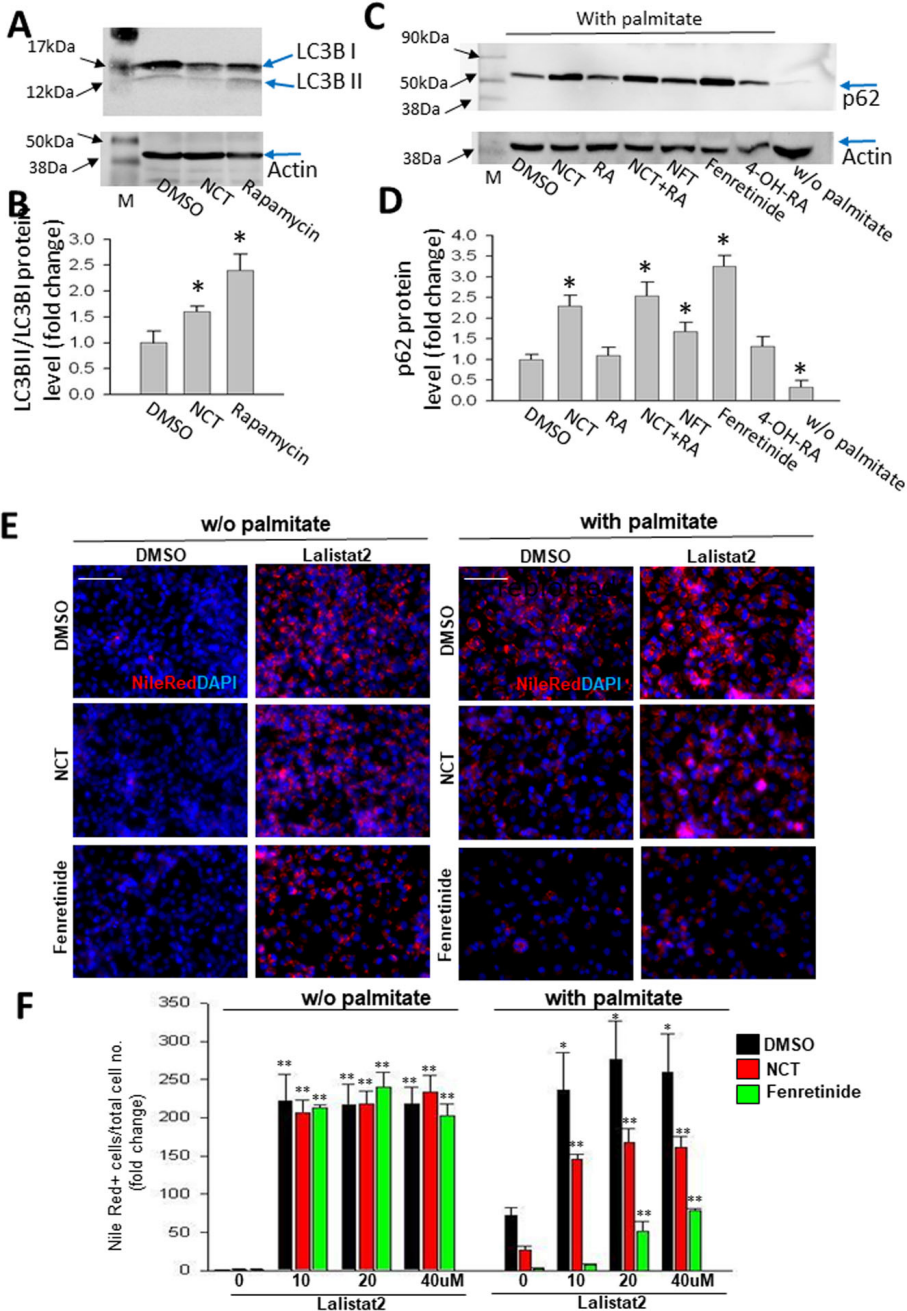
NCT promotes fat clearance by inducing lipophagy. T6PNE cells were treated with 0.25 mM palmitate for 2 days with the indicated compounds, followed by harvesting for Western blot and imaging. **A** Anti-LC3B Western blot demonstrating an increased ratio of LC3B II to LC3B I. For Western blotting, T6PNE cells were treated with DMSO (lane 1), NCT (10μM), rapamycin (10μM) and blotted with LC3B antibody. After detecting LC3B or p62 (panels **A**, **C**), the same membrane was reblotted with anti-β-actin antibody to ensure equal amounts of protein in each lane. **B** Quantification of ratio in LC3B II to LC3B I. **C** p62 Western blot with T6PNE cells treated with NCT (10μM), RA (10μM), NCT + RA (each at 10 μM), NFT (10 μM), fenretinide (5 μM), 4-OH-RA (20 μM), or without palmitate. **D** Quantification of p62 protein expression normalized to actin. The same membrane was reblotted with anti-β-actin antibody to ensure equal amounts of protein in each lane. Fenretinide had a statistically significant effect but retinoic acid did not. **E** Lysosomal acid lipase is required for the effect of NCT on fat clearance. Representative images of T6PNE cells treated for 2 days with or without palmitate, NCT (5 μM), fenretinide (5 μM) and the LAL inhibitor Lalistat2 (20 μM), followed by staining with Nile Red to visualize intracellular fat. **F** Quantification of the conditions shown in panel E. Values represent the mean ± SE of 3–4 biological replicates. All fold changes were calculated relative to 0μM Lalistat2 in the absence of palmitate, *p < 0.05, **p < 0.01 (DMSO vs. NCT or each NCT vs NCT + Lalistat2). Scale bar = 100 μm.

The ability of dihydroceramides to effect fat clearance from cells raised the question of the mechanism by which they act. Some studies have found a role for dihydroceramides in autophagy^34^. Autophagy involves alterations in LC3B through lipidation and effects on the level of the p62 autophagy receptor that can be monitored by Western blotting, with the polarity of those changes being complex and varying under different circumstances and in different cells^35,36^. Contrary to the classical situation in which p62 varies inversely with autophagic flux, but similar to rapamycin, which was used as a positive control to induce autophagy^37^, NCT induced increases in the LC3B-II to LC3B-I ratio (Fig. 5A, quantified in B), and in p62 (Fig. 5C, quantified in D).

Lipophagy is a form of autophagy that has been associated with dihydroceramides^20^. A central aspect of lipophagy is the cleavage of triglycerides from lipid droplets by lysosomal acid lipase (LAL)^20^. Direct association between lipid droplets and lysosomes has been demonstrated^38^. To determine whether lipophagy plays a role in fat clearance induced by NCT, we used the LAL inhibitor Lalistat 2^39^. Consistent with NCT acting by stimulation of lipophagy, Lalistat 2 inhibited the ability of NCT to promote fat clearance (Fig. 5E, quantified in F, Supplementary Fig. 5).

### NCT reverses hepatic steatosis

Having established that NCT reduces the level of stored fat in cells in vitro, it was of interest to determine its effect in vivo. We focused on the liver, the organ expressing the highest level of HNF4α and a major site of pathological fat storage, i.e., NAFLD. HNF4α has been recognized as playing an important role in NAFLD^40^. Adipose cells, the other major site of fat storage, do not express HNF4α. NCT was administered by IP injection (200 mg/kg bid) for two weeks to C57BL/6 J DIO mice maintained on a 60% fat calorie diet. The dose was selected on the basis of a dose-response study in which mice were injected with NCT at increasing doses (30, 60, 120 and 240 mg/kg bid for 3 days), which was well tolerated (Supplementary Table 1).

**Fig. 6:**
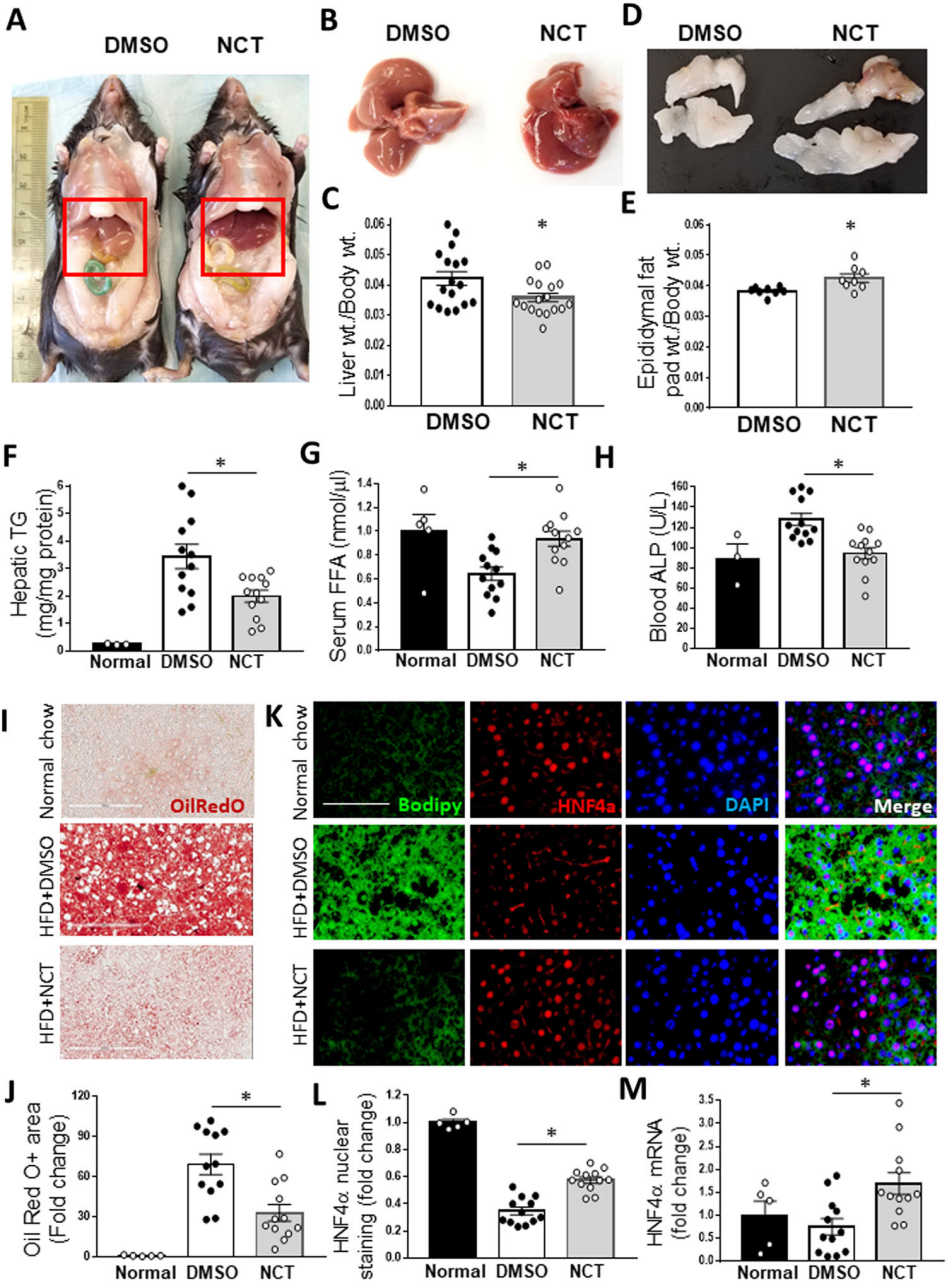
NCT reverses hepatic steatosis in vivo. DIO mice (C57BL/6 J) were injected intraperitoneally with NCT (200 mg/kg bid) for two weeks, followed by harvesting of organs. **A** Red box indicates the area of the liver, demonstrating a marked difference in color. **B** Dissected liver from representative mice indicating difference in color and weight (quantified in **C**, *N* = 12 for each group). **D** Epididymal fat pads from representative mice showing increased weight with NCT (quantified in **E**, N = 12 for each group). **F** Hepatic triglyceride (TG) content normalized to hepatic protein (Normal chow control, *N* = 3, DMSO and NCT, *N* = 12). **G** Serum free fatty acid (FFA) level (Normal chow control, *N* = 5 and DMSO and NCT, *N* = 12). **H** Blood alkaline phosphatase (ALP) level (Normal chow control, *N* = 3 and DMSO and NCT, *N* = 12). **I** Representative photomicrograph of hepatic Oil Red O staining (scale bar = 200 μm). **J** Oil Red O quantification. The percent of the liver section positive for Oil Red O was measured using Image J with a consistent threshold setting and normalized to liver sections from mice fed normal chow. **K** Representative liver sections stained for Bodipy (green), HNF4α (red), DAPI (blue) and merged images in mice fed normal chow or HFD plus DMSO or NCT. **L** Quantification of HNF4α nuclear staining. HNF4α nuclear staining intensity with non-specific cytoplasmic staining from same cell subtracted. Normalized to livers from mice fed normal chow. **M** Quantification of hepatic HNF4α mRNA level. Dots indicate individual mice. Values represent the mean ± SE, Normal chow control, *N* = 3–5; DMSO and NCT, *N* = 12. *p < 0.05, **p < 0.01. Scale bar = 100 μm.

After two weeks, the livers of the NCT-injected mice exhibited a shift in the color of the liver from yellow to red, as expected with a decrease in fat content (Fig. 6A, B). Livers from NCT-injected mice weighed less than those of the control mice, as expected if NCT was stimulating loss of hepatic fat (Fig. 6C) and this was borne out by a reduction in the hepatic triglyceride content (Fig. 6F). Analysis of liver sections revealed a decrease in stored fat by Oil Red O staining (Fig. 6I, J). Control and NCT-injected mice gained weight to a similar degree and both groups remained healthy and active (Supplementary Fig. 6).

The lack of a difference in body weight combined with the decreased liver weight implied that fat released from the liver must be present elsewhere. Consistent with that, there was an increase in epididymal fat pad mass (Fig. 6D, E). The shift of fat from the liver to adipose tissue suggested that fatty acids had to have traveled through the circulation to shift from one tissue to another. This would be expected if lipophagy was being induced by NCT, as lipophagy involves the release of fatty acids from cells through the action of LAL, which should lead to an increase in circulating free fatty acids (FFA). To test that prediction, we employed a protocol used in humans^41^ and mice^42^ in which a glucose challenge is used to stimulate insulin secretion, which inhibits FFA release from adipocytes, leaving liver-derived FFA as the major source of circulating FFA. Consistent with our hypothesis, NCT induced an increase in free fatty acids (Fig. 6G). Serum TG was increased by NCT at week 1 of NCT administration but not at week 2, the end of the study (Supplementary Fig. 7A).

### ALP was decreased by NCT

Markers of liver injury are elevated in NAFLD, including alkaline phosphatase (ALP) ALP has been studied as an early indicator of the transition to hepatic fibrosis as part of the progression from NAFLD to non-alcoholic steatotic hepatitis (NASH)^43,44^. Mice treated with NCT exhibited decreased ALP (Fig. 6H). There was no change in the level of other markers in the VetScan panel^45^ (Supplementary Fig. 7B).

### NCT reverses negative effects of fatty acids on HNF4*α* expression

HNF4α feeds back on its own promoter in a positive feedback loop^23,46,47^ and is the best marker of HNF4α activity in our experience^7,8^. In vitro, NCT and NFT induced *HNF4α* expression in T6PNE cells (Fig. 1G) and primary human hepatocytes (Supplementary Fig. 8). In vivo, we showed previously that the potent HNF4α antagonist BI6015 caused loss of *HNF4α* expression in the liver^8^, so we tested whether NCT had an effect in vivo. In control DIO mice, HNF4α protein was decreased compared with mice on a normal chow diet, as expected given our previous finding that fatty acids inhibit HNF4α activity^8^ (Fig. 6K, L). Interestingly, we did not detect a decrease in HNF4a mRNA with HFD (Fig. 6M), which could be due to post-translational regulation of HNF4α^48^. NCT reversed the loss of HNF4α protein (Fig. 6K, L) and mRNA (Fig. 6M).

### CYP26A1, an HNF4*α* downstream target, plays an important role in the induction of fat clearance

In the mouse pancreas and T6PNE cells, NCT induced *SPNS2* expression (Supplementary Figure 9), which is required for the reversal of cellular steatosis (Fig. 3). However, NCT did not induce a change in *SPNS2* expression in the mouse liver (Supplementary Fig. 9) or primary cultured human hepatocytes (Supplementary Fig. 8). Because we deemed it unlikely that NCT was acting by a fundamentally different mechanism to clear fat from the liver than in T6PNE cells in vitro which was originated from human pancreas, we examined for other genes induced by NCT in the liver that might be involved in the same pathway (GSE172234, Supplementary Fig. 10). *CYP26A1* is induced by HNF4α in conjunction with retinoic acid (RA)^49^ and converts RA to multiple metabolites, including 4-oxo-RA, 5,6 epoxy-RA, and 4-OH-RA^50^. RA is synthesized in the liver and so is abundant there^51^.

**Fig. 7:**
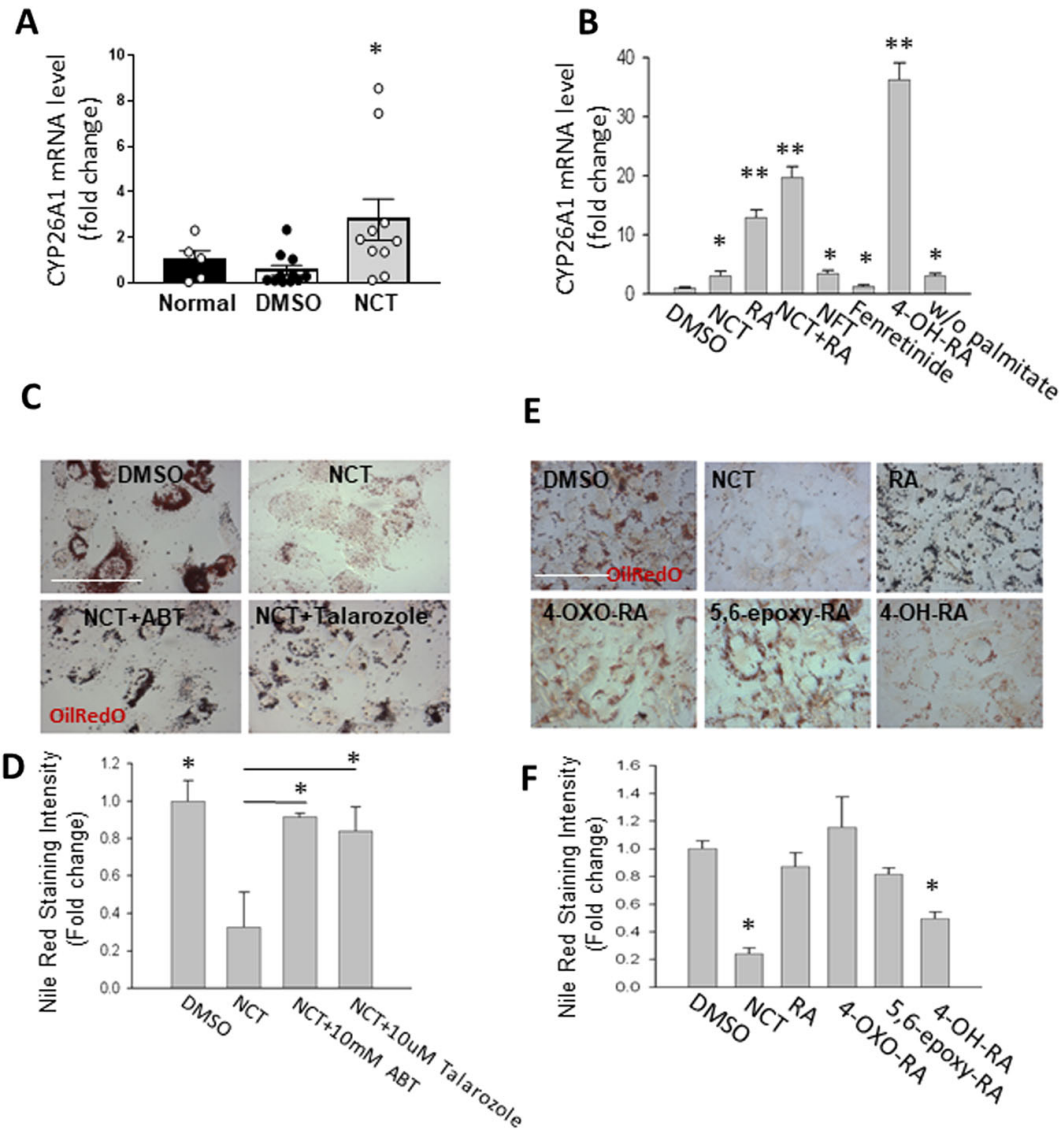
Fat clearance by NCT acts through CYP26A1. **A** RT-PCR analysis of hepatic CYP26A1 mRNA level (Normal chow control; *N* = 5 and for DMSO and NCT; *N* = 10). **B** RT-PCR analysis of CYP26A1 mRNA level in T6PNE cells treated for 2 days with DMSO, NCT (10 μM), RA (10 μM), NCT + RA (10 μM), NFT (20 μM), fenretinide (5 μM), 4-OH-RA (20 μM) on 0.25 mM palmitate and DMSO w/o palmitate. **C** CYP26A1 is required for fat clearance by NCT. T6PNE cells treated with palmitate (0.25 mM) plus the indicated compounds for 2 days, including the inhibitors ABT (10 mM, broad CYP inhibitor) and Talarozole (10uM, selective CYP26 inhibitor). **D** Quantification of the effect of CYP inhibitors on fat clearance by NCT (vs NCT for significance). **E** Metabolites from CYP26-mediated RA metabolism induce fat clearance. Representative images of T6PNE cells treated for 2 days with NCT or the RA metabolites 4-OXO-RA, 5,6-epoxy-RA, or 4-OH-RA (20μM) and stained with Nile Red. **F** Quantification of effect of RA metabolites on fat clearance. Values represent the mean ± SE of 4 biological replicates, *p < 0.05, **p < 0.01 (vs DMSO). Scale bar = 100 μm.

As we had shown previously that fenretinide, a synthetic retinoid that inhibits DES1, induced fat clearance from T6PNE cells, we hypothesized that CYP26A1 was a good candidate for playing a role in NCT-induced fat clearance from the liver. NCT induced *CYP26a1* in the mouse liver (Fig. 7A), cultured human hepatocytes (Supplementary Fig. 8) and in T6PNE cells (Fig. 7B). The generalized CYP inhibitor ABT^52^ and the specific CYP26 inhibitor talarazole^53^ inhibited fat clearance by NCT (Fig. 7C, D).

Having demonstrated a role for CYP26A1 in fat clearance downstream of NCT, we hypothesized that one of the retinoic acid metabolites produced by CYP26 should induce fat clearance. Consistent with that, 4-OH RA induced fat clearance from T6PNE cells (Fig. 7E, F). Interestingly, 4-OH-RA also induced an increase in *CYP26A1* mRNA (Fig. 7B) Thus, RA and its metabolites play important roles in fat storage in the liver through transcriptional and post-transcriptional mechanisms, respectively (Supplementary Fig. 1).

### NCT induces dihydroceramide production

**Fig. 8:**
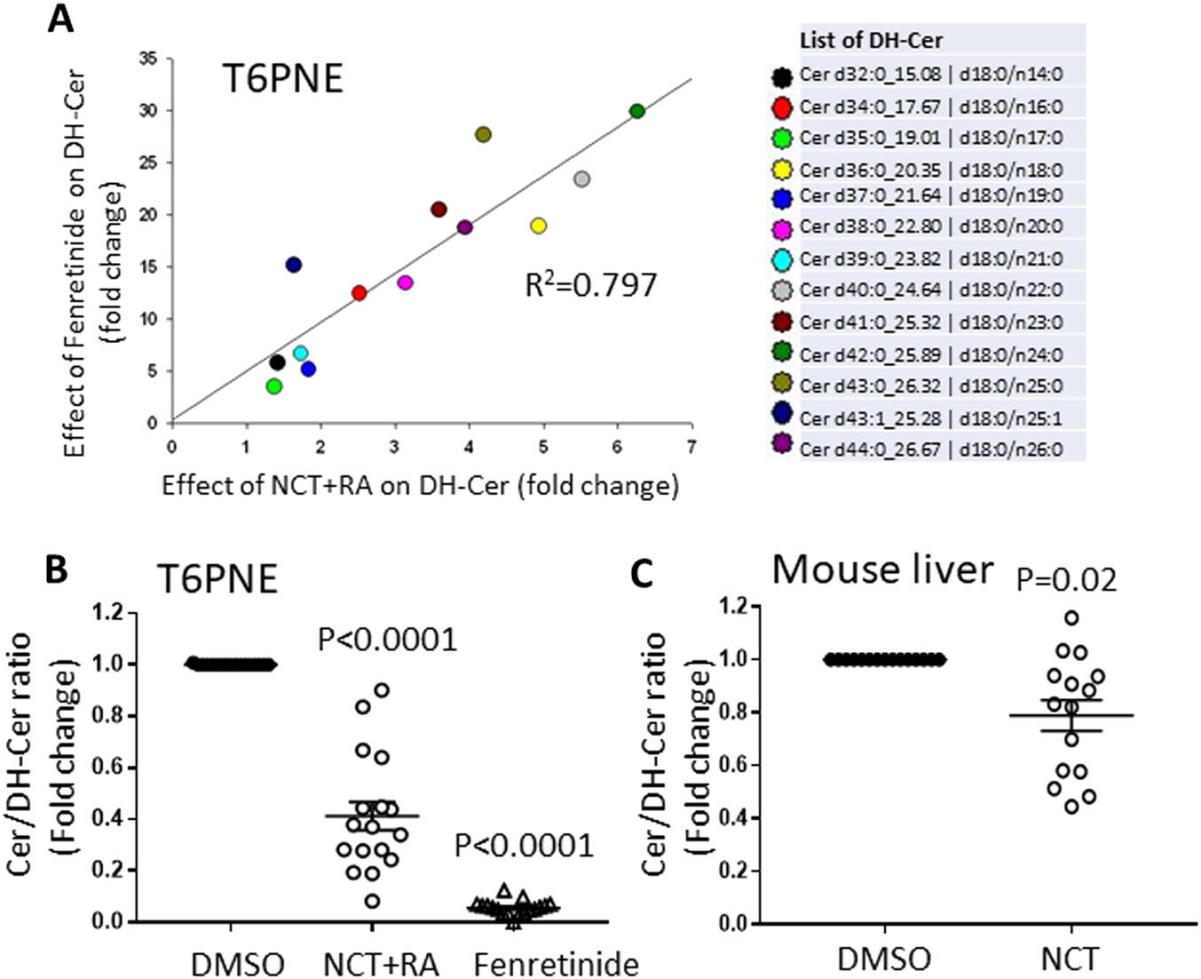
Dihydroceramides were increased in T6PNE cells and mouse liver by NCT. Ceramides and dihydroceramides were measured in vitro and in vivo following treatment with the indicated compounds. **A** T6PNE cells were harvested 2 days after treatment with DMSO, NCT + RA (10μM) and Fenretinide (5μM). NCT and fenretinide induced multiple identical dihydroceramides (*R*^2^ = 0.797). **B** The ceramide (Cer)/dihydroceramide (DH-Cer) ratio was decreased in T6PNE cells treated with NCT + RA or Fenretinide. **C** Livers from mice treated with DMSO or NCT for 2 weeks. The ceramide/dihydroceramide ratio decreased in response to NCT (*P* = 0.02, see Supplementary Tables 2 and 3 for individual ratios and raw data). *N* = 3–4 biological replicates (vs DMSO). Note that C17 fatty acids are derived from consumption of plants and are not produced in mammals.

A key prediction of our model of the control of hepatic fat storage by HNF4α is that HNF4α should increase the production of dihydroceramides (Supplementary Fig. 1). This was tested in vitro and in vivo using a lipidomic approach^54^. Lipidomic analysis revealed that multiple dihydroceramides were increased by NCT in T6PNE cells. Strikingly, there was a strong correlation between the dihydroceramides produced in response to NCT and those induced by fenretinide (Fig. 8A, *R*^2^ = 0.79), consistent with the model (Supplementary Fig. 1) that a downstream effect of NCT is to inhibit DES1. Both NCT + RA (used to induce CYP26A) and fenretinide induced a substantial decrease in the ratio of ceramide produced by the action of DES1 relative to the corresponding dihydroceramide (Fig. 8B, Supplementary Tables 2 and 4). This was evident as well in lipidomic analysis of livers from mice injected IP with NCT (Fig. 8C, Supplementary Tables 3 and 5). This is consistent with NCT administration in vivo resulting in inhibition of DES1 and consequent increased dihydroceramide production in the liver as predicted by our model (Supplementary Fig. 1).

## Discussion

Understanding the regulation of fat storage in the liver is vital to developing treatments for fatty liver disease, a major public health problem^3^. Here, we report the discovery of a previously unknown pathway by which HNF4α controls hepatic fat storage. That discovery was made possible by the discovery of potent HNF4α agonists, also reported here. Stimulation of HNF4α activity with the HNF4α agonist NCT led to reversal of hepatic steatosis in the short time frame of two weeks. Encouragingly for its therapeutic potential, NCT treatment was completely nontoxic and led to a decrease in ALP, an important marker of liver injury and progression from NAFLD to NASH. ALP has been put forth as a marker of hepatic fibrosis^43^ and is also used commonly as a biomarker for biliary cholangitis^55,56^.

The HNF4α agonists described here are structurally similar to alverine and benfluorex, known drugs that we found previously to be HNF4α activators^7^. The strategy of drug repurposing that we began with is being used for many diseases, including COVID-19^57^, but there are few examples of success^58^. Alverine and benfluorex are weak HNF4α activators and are unsuitable for in vivo use, but they served as an important starting point for the efforts described here, and thus can be taken as partial validation of the strategy. However, NCT and NFT are much more potent, making possible in vivo and mechanistic studies. NCT and NFT are found in plants, including some consumed by humans. They have no known role as nuclear receptor ligands or mediators of plant metabolism, and there is no HNF4a homolog in plants. There have been many reports of compounds found in plants and plant extracts that have beneficial effects on disorders caused by fat excess, including type 2 diabetes and fatty liver disease^59,60^.

NCT and NFT are found in association with plant cell walls. They are induced in response to damage and are thought to play a role in pathogen defense^18^. However, little is known about their function. In animal cells and in mice, they have been shown to have anti-inflammatory properties^61^. The concentration of NCT in most plants is low, as it is an NFT precursor, being converted to NFT by O-methylation of the 3-hydroxyl group of the phenylpropenic acid moiety^18^. NFT is more abundant, being found in various plants at a few tens of micrograms per gm of dry plant, but that will vary depending on the degree to which the compounds were induced prior to harvest^62^. Given their poor oral bioavailability and low abundance in plants that are commonly consumed as food, NCT and NFT are unlikely to be physiologically relevant sources of HNF4α ligands in most human diets, but that is a question worthy of additional investigation.

The mechanism that we found for the control of hepatic fat storage by HNF4α involves the induction of lipophagy, a form of autophagy^20^. Genetic knockout studies and molecules that stimulate autophagy have implicated autophagy in fatty liver disease^63^. However, the induction of lipophagy by HNF4α has not been suspected previously. An advantage of stimulating lipophagy by HNF4α agonists rather than by general stimulators of autophagy^64^ is that HNF4α expression is highly tissue restricted, being expressed predominantly in the liver, pancreas, kidney and intestine. We did not observe any systemic effects of NCT, and in fact were unable to establish a maximum tolerated dose because of the lack of toxicity. The fat released from the liver appeared to be taken up by adipocytes, which do not express HNF4α.

HNF4α is a nuclear receptor transcription factor, so we hypothesized that the mechanism by which it stimulates lipophagy must involve HNF4α-mediated transcriptional effects on genes that ultimately promote lipophagy. In the T6PNE cells used in the insulin promoter assay, upregulation by NCT of SPNS2, a transporter, led to the identification of dihydroceramides as playing a key role in stimulating lipophagy downstream of HNF4α. In the liver in vivo, HNF4α acts in conjunction with retinoic acid to activate CYP26A1 transcription. In one of the many feedback loops in this system, RA is then metabolized by CYP26 itself to a number of metabolites^65^, of which we found at least one, 4-OH RA, to inhibit the dihydroceramide metabolizing enzyme DES1 to increase the production of dihydroceramides. 4-OH RA also induces *CYP26A* expression. Complex and interacting feedback loops appear to be a central feature of the pathway described here. One of the most important of those is the inhibition by palmitate of HNF4α activity, which should lead to decreased lipophagy and consequent increased fat storage. However, de novo dihydroceramide synthesis begins with palmitate, the major fat consumed by humans and a major effector of lipotoxicity^26^. We show here that dihydroceramides stimulate lipophagy, resulting in decreased fat storage, opposing its negative effect on HNF4α as a direct inhibitor through binding to HNF4α^8^ (Supplementary Fig. 1).

Dihydroceramides have been implicated in lipotoxic diseases, including hepatic steatosis and type 2 diabetes^34^. They have been measured in the blood type 2 diabetes and cardiovascular disease^66^. Genetic ablation of DES1 improves insulin resistance and hepatic steatosis^67^, but dihydroceramide function in those diseases has not been well understood. Under our model, the highest concentration of secreted dihydroceramides will be in the immediate vicinity of their site of export by SPNS2, so it is likely that they act primarily in an autocrine and/or paracrine manner through S1PRs on nearby cells. We found that S1PR3 is necessary for the action of NCT and dihydroceramides in T6PNE cells and so must be a receptor for dihydroceramides, but the other four receptors have not been studied in this regard. They are expressed in complex patterns, which could potentially contribute to effects in other tissues. For example, dihydroceramides play important roles in hematopoietic stem cells^68^, so restricting activity primarily to the organs that are affected by a particular disease such as NAFLD by targeting HNF4α could potentially avoid undesirable side effects. An interesting area for future studies will be to determine how S1P receptors signal to lipid vesicles to promote lipophagy.

The model described above does not require the existence of an endogenous HNF4α agonist, and no such agonist has been found. Rather, HNF4α appears to exhibit a high level of basal activity in the absence of ligand binding, with fatty acids acting as endogenous antagonists^14^. The high potency of NCT as an HNF4α agonist allows for activation of lipophagy even in the face of a high, pathological level of endogenous fat. The finding that fatty acids regulate HNF4α activity combined with the finding that HNF4α regulates lipophagy support a model in which HNF4α is repressed by high levels of ingested fat under fed conditions. This leads to suppression of hepatic lipophagy and storage of any available fat. Under the condition of starvation, HNF4α will not have bound fat, as found for linoleic acid^14^, and thus will be more active, leading to increased hepatic lipophagy and release of stored fat. Ingestion of a high level of dietary fat will lead to constitutive downregulation of HNF4α activity and thus to hepatic steatosis.

In addition to hepatic steatosis, HNF4α is implicated in a number of other diseases^69^, that affect tissues with high HNF4α expression, including type 2 diabetes, where HNF4α has been found to be a type 2 diabetes gene in a number of GWAS studies^70^. Haploinsufficiency for HNF4α causes MODY1, a monogenic form of diabetes^71^. Thus, HNF4a agonists could be of benefit in other settings in addition to NAFLD. Of note, hepatic steatosis and consequent hepatic insulin resistance play an important role in T2D pathogenesis, so ameliorating NAFLD could have beneficial effects on diabetes independent of any effects on the islet^72^. In the intestine, HNF4α has been implicated in inflammatory bowel disease by GWAS studies^73^. A site of significant HNF4α expression is the kidney, and obesity is a strong risk factor for the development of renal disease^74^. Thus, pharmacologic activation of HNF4α may be useful in diseases affecting organs other than the liver. The HNF4α activators studied here, NCT and NFT, differ in their respective abilities to activate the insulin promoter (Fig. 1). Thus, it may be desirable to screen for additional compounds to find those with optimal activity for each potential disease target.

## Materials and methods

### Compound screening

The T6PNE insulin promoter assay has been described previously^10^ and was performed here with slight modifications as follows: T6PNE cells were seeded at 2000 cells per well in 384-well tissue culture plates (Greiner Bio-One) in the presence of 0.5 µM tamoxifen. Compounds listed in Fig. 1A in DMSO were dispensed with an Echo 555 Acoustic Liquid Handler (Beckman Coulter). Three days after compound addition, cells were fixed in 4% paraformaldehyde (USBio) for 15 min and stained with DAPI (0.167 µg/ml, Invitrogen). Blue (DAPI) and green (GFP) channels were imaged using a Celigo imaging cytometer (Nexcelom Bioscience). The number of GFP-positive cells was normalized to the DAPI-positive cell number and fold change calculated relative to the DMSO control.

### Cell culture

T6PNE cells were maintained in RPMI (5.5 mM glucose, Corning) supplemented with 10% fetal bovine serum (FBS, Sigma-Aldrich) and 1% penicillin-streptomycin (pen-strep, Gibco). Cells were maintained in 5% CO_2_ at 37 °C. For the insulin promoter assay, 0.5 µM tamoxifen (Sigma-Aldrich) was added to T6PNE cell culture media. HepG2 or HeLa cells were cultured in DMEM (high glucose, Corning) supplemented with 10% FBS and 1% pen-strep and maintained at 5% CO_2_, 37 °C.

### Lipid staining (in vitro) and analysis

Oil red O and Nile Red staining were used to measure lipid accumulation. Oil Red O staining was done as described^75^. Briefly, fixed cells were incubated with Oil Red O solution (Poly Scientific) for 3 h, followed by photomicrography (Olympus, IX71). For Nile Red staining, dye (1: 500 in PBS from 1 mg/ml in ethanol stock, Sigma) was added for 30 mins and DAPI added for 10 mins at room temperature. Quantification of Nile Red staining was done with a Celigo imaging cytometer (Nexcelom Bioscience). More than 4000 cells were analyzed for each quantification. The number of Nile Red-positive cells was normalized to the DAPI-positive cell number for each well and fold change was calculated relative to a DMSO control well.

### Oil Red O staining (in vivo) and analysis

Slides containing frozen liver tissue sections from mice were air dried for 10–20 min followed by rehydration in distilled water. Sections were immersed in absolute propylene glycol (Cat# 151957, MP Biomedicals, LLC, USA) for 2 min followed by 0.5% in oil red O solution (Cat#K043, Poly Scientific R&D, USA) for 2 h. Slides were then differentiated in 85% propylene glycol solution, washed with dH_2_O for 2 h, and mounted using glycerin jelly mounting medium. All slides were scanned at a magnification of 20x using the Aperio Scanscope FL system (Aperio Technologies Inc., Vista, CA, USA). The liver area stained with oil red O was measured using image J software as described^75^, with some modifications. Oil red O-stained liver images were opened in Image J software. Using the *Analyze* > *Set Scale* command, the scale bar of the images was set to 200 um. RGB images were then converted into gray scale images using the *Image* > *Type* > *RGB Stack* command and were split into red, blue and green channels. Using the *Image* > *Adjust* > *Threshold* command, the threshold was manually set to highlight the oil red O-stained lipid droplets in the green channel. We used the same threshold for all the images in all treatment groups and the % oil red O-stained area was obtained using the *Analyze* → *Measure* tool command. Fold change was calculated by normalizing the values to images from mice fed normal chow.

### Palmitate–BSA complex

Palmitate (150 mM) (Sigma–Aldrich) was prepared in 50% ethanol and precomplexed with 15% fatty acid-free BSA (Research Organics, Cleveland, OH, USA) in a 37 C water shaker. BSA-precomplexed palmitate was used as a 12 mM stock solution for all assays with a final concentration of 0.25 mM palmitate in cell culture medium.

### siRNAs

siRNAs were purchased from Ambion. For transfections, 24 μL of each siRNA (1 μM stock) was mixed with 90 μL of a 1:100 dilution of Lipofectamine RNAi MAX (Invitrogen, Waltham, MA, USA) in Opti-MEM. Transfection was done in 24 well plates (Thermo Fisher Scientific, Waltham, MA, USA), by incubation with cells for 30 min. at room temperature. One day after transfection, cells were transferred to 96 well plates (2000 cells per well) and incubated at 37 °C for an additional day. Forty-eight hours after transfection, either palmitate-BSA complex or BSA control plus or minus compounds was added for 2 days. For validation of siRNAs, transfected cells were harvested for RNA purification and QPCR for each gene 2 days after transfection.

### Q-PCR

Quantitative PCR (Q-PCR) from in vitro cell culture experiments used total RNA purified using RNeasy Kits (Qiagen). For liver tissue samples, total RNA was isolated using Trizol (Invitrogen) and prepared for RNAseq (mouse liver DMSO and NCT, *N* = 3). cDNA was amplified using 3 μg of total RNA using qScript cDNA SuperMix (Quanta BioSciences, Beverly, MA, USA). Q-PCR analysis was performed using SYBR® Select Master Mix (Applied Biosystems) and ABI 7900HT (Applied Biosystems, Thermo Fisher Scientific). The Ct values of mRNA expression were then normalized to the 18 s rRNA values and are expressed as fold change over samples from mice fed normal chow for in vivo experiments or DMSO controls for cell culture experiments.

### DARTS assay

This was conducted as described previously^7^. HepG2 cells were treated with DMSO, BI6015, NCT, NFT at a concentration of 40 or 80 µM for 16 hr. Total cell protein was extracted and measured by BCA protein assay (Thermo Scientific). Each sample was split into two aliquots for proteolysis without (−) or with (+) Subtilisin (Sigma-Aldrich). Forty mg of cell lysate was incubated with or without protease (40 ng/ml subtilisin) for 35 min at room temperature.

### Western blotting

Whole-cell extracts were prepared by incubation in RIPA buffer (Invitrogen) containing protease inhibitors (Calbiochem, San Diego, CA). Protein (40 mg) was separated on 12% or 16% Tri-Glycine gels (Invitrogen) and transferred to Immobilon P membrane (0.2 μm pore, Millipore). After 1 h in phosphate-buffered saline–Tween (PBST) with 3% milk, membrane was incubated with antibodies to HNF4α (mouse, Novus, 1:1000), LC3B (rabbit, Novus, 1:500), p62 (SQSTM1, mouse, Santa Cruz, 1:1000) or β-actin (mouse, Santa Cruz, 1:2000), followed by secondary antibody conjugated to horseradish peroxidase (1:5000, Jackson Immune). Signal was revealed by ECL (Thermo) and imaged with a ChemiDoc MP imager (Bio-Rad).

### BCA assay

The bicinchoninic acid (BCA) protein assay kit assay (Thermo scientific) was used to measure protein amount for DART, Western blotting and TG assays. Absorbance at 550 nm was determined using a plate reader.

### Mice

12-week-old C57BL/6 J DIO male mice (cat#380050) were purchased from Jackson Laboratory and were fed with high fat diet containing 60 kcal% fat (Research Diets cat #D12492). Mice were maintained in a 12-h light/day cycle. After 2 weeks of acclimation, mice with similar body weights were randomly assigned to treatment or control groups.

For dose response experiments, mice were injected intraperitoneally with10% DMSO as vehicle control or 4 different doses of NCT (30, 60, 120 and 240 mg/kg of body weight) dissolved in 10% DMSO. Mice received 2 doses per day with a 5 h interval between injections for 3 days. Mice were observed for any adverse effects for a week of post treatment as shown in Supplementary Table 1.

To test the effect of NCT (Sundia MediTech Company, Ltd., Custom synthesis), 200 mg/kg was injected IP bid for 14 days. On day 15, mice received a final dose of NCT followed by IP injection with 3 g/kg dextrose. One h later, blood samples were collected and mice were euthanized using pentobarbital. Total mouse, liver and epididymal fat pad weights were measured. Dissected liver samples were washed in cold PBS, cut into small pieces and distributed for analyses. For RNA isolation and ELISA, liver samples were snap frozen using liquid nitrogen and stored at −80 °C. For histomorphometry and immunofluorescence analysis, liver samples were fixed in 4% of cold PFA and processed for histology. All animal experiments were approved by the Institutional Animal Care and Use Committee (IACUC) of the Sanford Burnham Prebys Medical Discovery Institute in accordance with national regulations.

### Immunofluorescence and analysis

Frozen liver sections were permeabilized using 0.3% Triton-X and incubated in antigen retrieval solution (Antigen retrieval citrate, Biogenex) at sub boiling temperature for 10 min. Subsequently, sections were incubated with blocking buffer containing 5% normal donkey serum (Jackson Immuno Research) followed by incubation overnight at 4 °C with mouse monoclonal primary antibody against HNF4 (1:800, Cat# PP-H1415-00, R&D Systems). Sections were washed and incubated with anti-mouse secondary antibody coupled with Alexa flour 488 (1:400, Invitrogen) or with DyLight 647 (1:400, Jackson Immuno) for 1 h at room temperature and counterstained with DAPI (40,6-diamidino-2- phenylindole, Sigma Aldrich). For lipid droplet staining, slides were incubated in Bodipy 500/510 (1:100 from 1 mg/ml, 4,4-Difluoro-5-Methyl-4-Bora-3a,4a-Diaza-*s*-Indacene-3-Dodecanoic Acid, Invitrogen) for 30 min. Slides were mounted using fluorescence mounting medium and images were obtained at 40x magnification using an Olympus IX71 fluorescence microscope. Fluorescence intensity of HNF4α-stained nuclei was calculated using MetaMorph TL software (version 7.6.5.0, Olympus).

### Free fatty acid quantification

The serum FFA level was measured using the Free Fatty Acid Quantification Colorimetric/ Fluorometric Kit (Cat #K612, BioVision, USA). Fold change was calculated by normalizing to values from mice fed normal chow.

### Triglyceride analysis

Serum and liver TG level was measured using the Triglyceride Calorimetric Assay Kit (Cat# 10010303, Cayman Chemicals, USA). Fold change was calculated by normalizing the values from normal chow mice.

### Liver profile analysis

100 μL of whole blood was collected in lithium heparin blood collection tubes and transferred to single use VetScan mammalian liver profile reagent rotors. The levels of multiple analytes present in the blood samples were quantified using a VetScan VS2 Chemistry Analyzer (Abaxis North America, USA).

### Serum alkaline phosphatase analysis

The alkaline phosphatase level in serum samples was quantified using a Catalyst One Chemistry Analyzer (IDEXX Laboratories, Inc. USA).

### Microsomal stability

Microsomal stability studies were performed in the Conrad Prebys Center for Chemical Genomics. In vitro metabolism was conducted in a system consisted of NADPH generating system, test compound, and Tris·Cl buffer. The mixture was pre-incubated at 37 °C for 30 min. Reactions were initiated by addition of mouse or human liver microsomes suspension and shaken at 37 °C with air exposure. To generate the stability curve for the test compound, the incubation was terminated at 0, 5, 15, 30 and 60 min. NFT and NCT concentrations were determined by LC-MS. The result of metabolic stability was expressed as the percentage of compound remaining at 1 hr. The in vitro half-life (t1/2) and intrinsic clearance (Clint) were calculated based on drug depletion over incubation time.

### Pharmacokinetics

Murine PK was conducted by WuXi AppTec (Shanghai, China). C57BL/6 male mice of age 7-9 weeks were obtained from SLAC Laboratory Animal Co (Shanghai, China). Mice were fasted for 12 h prior to compound administration. Oral gavage was used for PO dosing. For IV dosing, compound was administered by tail vein injection. For compound concentration determination, 25μL of blood was collected from the submandibular or saphenous vein and processed for plasma. Plasma concentration of compound was determined by LC-MS/MS.

### Lipidomics (Ceramide panel)

Ceramides and dihydroceramides were measured in the UCSD Lipidomics Core Facility as previously described (Quehenberger et al.^54^). Briefly, samples were extracted using the butanol: methanol (BUME) method^76^. The lipid layer was collected and run on a Thermo-Vanquish UPLC (Thermo Scientific) with a Cortecs T3 (C18), 2.1 mm×150 mm; 1.8 uT3 column and a binary solvent system. Mass spectrometry employed a Thermo Q Exactive instrument with MS/MS data dependent acquisition scan mode and LipidSearch software (Thermo Fisher Scientific). Lipid nomenclature is given for the validated species: Cer d32:1_19.14 | d18:1/n14:0. d stands for dihydroxy and t sands for tri-hydroxy; d32:1 indicates that the sum total carbons is 32 and the species contains 1 double bond; the number following the underscore is the retention time; d18:1/n14:0 indicates that indicates that the sphingoid base fatty acid is 18:1 and contains 2 hydroxy groups; 14:0 is the amide bonded fatty acid that contains no (n) hydroxy group; n14:0 indicates that 14:0 is the amide bonded fatty acid that contains no (n) hydroxy group.

### STRING network analysis

STRING (https://string-db.org) shows protein-protein interaction networks. The top 50 gene candidates upregulated in NCT treated mouse liver (*N* = 3) were analyzed. STRING functional enrichment analysis was also performed.

### Primary human hepatocytes

Experiments with primary human hepatocytes were performed by CN-Bio (Cambridge, UK). Primary human hepatocytes (PHHs), human Kupffer cells (HKs) and human stellate cells (HSCs) were seeded onto CN-Bio’s PhysioMimix LC12 MPS culture plates at 6 × 10^5^ cells for PHHs and 6 × 10^4^ cells for HKs and HSCs in 1.6 ml of CN-Bio’s HEP-lean media with 5% FCS. Throughout the experiment the cells were maintained at a flow rate of 1 μl/s. After 24 h (Day 1) of seeding, the media was changed to HEP-lean media and the cells were incubated until day 4 to allow the cells to form microtissues. At day 4 post seeding, media was changed to HEP-fat media and treated with DMSO or NCT (5, 15, 40 μM). Media was replaced on days 6 and 8. Cells were harvested on day 10 for RNA extraction.

### Statistical analysis

Data are presented as mean ± SEM of three or more samples as indicated. Statistical significance was assessed using Student’s *t*-test, ANOVA or *R*^2^ coefficient of correlation.

### Reagents

See Supplementary Table 6

## Supplementary information

CDDIS-21-0831R Supplementary Materials

## Data Availability

Unique reagents generated in this study will be made available upon request. An agreement with our institute’s Materials Transfer Agreement (MTA) may be required. Further information and requests for resources and reagents should be directed to Fred Levine (flevine@sbpdiscovery.org).
