# Supplementary material for: Liver fat storage is controlled by HNF4α through induction of lipophagy and is reversed by a potent HNF4α agonist
Source: Cell Death Dis. 2021 Jun 11;12(6):603. doi: 10.1038/s41419-021-03862-x (PMC8193211; doi:10.1038/s41419-021-03862-x)
Supplement: Supplementary file 1 — CDDIS-21-0831R Supplementary Materials [file 41419_2021_3862_MOESM1_ESM.docx]

Lee et al. Liver Fat Storage is Controlled by HNF4α Through Induction of Lipophagy and is Reversed by a Potent HNF4α Agonist

**SUPPLEMENTARY MATERIALS:**

**Supplementary Figure 1. Model of the control of hepatic fat storage by HNF4α.** The model that we are suggesting for the control of lipid storage by HNF4a begins with active HNF4α (in our case activated by the agonist NCT) in the nucleus promoting the expression of downstream genes, including *HNF4α*, *SPNS2,* and *CYP26a1*. Cyp26 metabolizes RA, leading to the production of 4-OH-RA that inhibits DES1 and activates *CYP26*. DES1 inhibition causes increased production of dihydroceramides, which are secreted through the action of SPNS2. Once outside the cell, dihydroceramides act on S1PRs to induce lipophagy, leading to the release of fatty acids and decreased hepatic fat storage.

**Supplementary Figure 2. Insulin promoter, estrogenic, PPARγ agonist, and fat clearance assays.**

**A:** Assay for estrogenic activity. This was done as in (8, 10) with tamoxifen as the positive control. All compounds were added at a concentration of 10μM. **B:** Assay for PPARγ agonist activity. This was done as in (8, 10) with the PPARγ agonist troglitazone as a positive control. NCT was weakly positive at a concentration greater than that used for the studies performed here. **C.** NCT (10μM), but not the PPARγ agonist troglitazone (10μM), induced fat clearance from T6PNE cells. Red is Nile Red staining and blue is DAPI. Values represent the mean ± SE of 4 biological replicates, *p<0.05, **p<0.01 (vs DMSO). Scale bar=100μM.

**Supplementary Figure 3. Representative images for data shown in Figure 3.** HNF4α siRNA decreased the number of GFP-positive T6PNE cells induced by NCT, NFT, NcisCT and alverine, demonstrating dependence on HNF4α for the effect of NCT and NFT. Imaging was done using a Celigo imaging cytometer. Green is GFP and blue is DAPI nuclear staining. Scale bar=500μM.

**Supplementary Figure 4. Validation of siRNAs.** T6PNE cells were transfected with siRNA to each target gene. Two days later, cells were harvested for RNA isolation. QPCR was performed and normalized to the level of 18s rRNA. All siRNAs induced a significant decrease in the level of the target mRNA. Values represent the mean ± SE of 3 technical replicates, *p<0.05, (vs scrambled siRNA for each gene).

**Supplementary Figure 5. Lalistat2 inhibits the effect of NCT on fat clearance.** Cells from the experiment shown in Figure 5E, F were harvested for TG quantification, supporting the Nile Red staining shown in Figure 5E, F. Values represent the mean ± SE of 3 biological replicates, *p<0.05, (vs DMSO).

**Supplementary Figure 6. NCT had no effect on the body weight of mice.** Body weight was measured at the initiation of the experiment (Day 0) and following 2 weeks of IP injection of DMSO and NCT. Values represent the mean ± SE (N=12 for each group).

**Supplementary Figure 7. Liver profiles on blood and serum TG level from mice injected with NCT**. **A:** Serum TG level increased after 1 week of NCT treatment but was unchanged from DMSO at 2 weeks. DMSO-treated mice exhibited continuous increases in serum TG level at 1 and 2 weeks. **B:** Determination of the markers of liver function shown here was done using a VetScan panel (39), as per the manufacturer. Values represent the mean ± SE, N=9 for each group, *p<0.05, (vs DMSO).

**Supplementary Figure 8. HNF4α and CYP26a1 mRNA is induced by NCT in primary human hepatocytes.** Human primary hepatocytes were seeded on Matrix with lean media (Day 0) and changed to high fat media plus DMSO or NCT (5, 15, 40μM) on Day 4. At Day 10, cells were harvested for RNA extraction. HNF4α and CYP26a1, but not SPNS2 mRNAs were significantly induced by NCT. Values represent the mean ± SE of 3 biological replicates, **p*<0.05 (vs DMSO).

**Supplementary Figure 9. *SPNS2* mRNA was induced by NCT in T6PNE and mouse pancreas but not mouse liver.** *SPNS2* qPCR was performed on cDNA from T6PNE cells, mouse liver, and mouse pancreas. The Ct value for SPNS2 amplification in pancreas-derived samples was 31 for mouse pancreatic tissue but was 23 for mouse liver, reflecting a much higher level of expression. Values represent the mean ± SE of 6-9 biological replicates, **p*<0.01(vs DMSO).

**Supplementary Figure 10: STRING network and enrichment analysis. A:** Diagram from STRING shows protein-protein interaction networks for top 50 upregulated gene candidates in NCT treatment from RNA sequencing (GSE172234,<https://www.ncbi.nlm.nih.gov/geo/query/acc.cgi?acc=GSE172234>) with mouse liver and HNF4a. 51 nodes represent 50 upregulated candidates and HNF4a that are linked by 61 edges. Colored lines between the nodes indicate different types of evidence for the interactions between proteins as shown in the legend. 4 Members of the cytochrome 450 (CYPs) family of enzymes such as CYP26a1, CYP2b10, CYP17a1 and CYP26b1 are among the top 50 candidates and are highlighted in red, with labelling of their corresponding degree of upregulation (fold change). **B:** STRING functional enrichment analysis for the top 50 upregulated candidates from RNA sequencing of mouse liver. Retinoic acid catabolic process from biological process and retinoic acid 4-hydroxylase activity from molecular function were identified.

**SUPPLEMENTARY TABLES**

**Supplementary Table 1: Maximum tolerated dose study in mice.** Mice were injected with NCT at increasing doses (30, 60, 120 and 240 mg/kg bid for 3 days, N=3). All doses were well tolerated.

**Supplementary Table 2. Modulation of ceramides and dihydroceramides (DH-Cer) in T6PNE cells.** Ceramide (white) and DH-Cer (yellow) levels (raw data) were determined by lipidomic analysis of T6PNE cells for Ceramides in the UCSD Lipidomics Core. The ratio of each ceramide and its corresponding dihydroceramide was calculated from the raw data, and the fold change in the ratio was calculated for NCT+RA or Fenretinide relative to DMSO. Both NCT+RA and Fenretinide induced a reduction in the Cer/ DH-Cer ratio (graph shown in Figure 8B). N=3.

**Supplementary Table 3. Modulation of ceramides and dihydroceramides (DH-Cer) in mouse liver.** Ceramide (white) and DH-Cer (yellow) levels (raw data) were determined by lipidomic analysis of mouse livers for Ceramides in the UCSD Lipidomics Core. The ratio of each ceramide and its corresponding dihydroceramide was calculated from the raw data, and the fold change in the ratio was calculated for NCT relative to DMSO. NCT induced a reduction in the Cer/ DH-Cer ratio (graph shown in Figure 8C). N=4.

**Supplementary Table 4. Quantification of ceramides and dihydroceramides inT6PNE cells** Ceramide panel data from T6PNE cells. Cells treated with NCT. Cells were harvested and analyzed by the UCSD Lipidomics Core to determine the levels of multiple ceramides and dihydroceramides. For lipid nomenclature, d stands for dihydroxy and t stands for trihydroxy. The number following d or t is the sum total carbons and the number following the colon is the number of double bonds. Thus, d18:0 indicates a dihydroceramide with 18 carbons and d18:1 is the corresponding ceramide that has been converted from d18:0 by the action of DES1. Mean ceramide levels from4T6PNE (N=3 for each group) are shown. Data were generated blindly by the UCSD Lipidomics Core.

**Supplementary Table 5. Quantification of ceramides and dihydroceramides in mouse liver.** Ceramide panel data from mouse liver. Mean ceramide levels from mouse livers treated with DMSO or NCT for 2 weeks (N=4 for each group). Data were generated blindly by the UCSD Lipidomics Core.

**Supplementary Table 6. Reagents**

| **Chemical** | **Company** | **NO. catalog** |
| --- | --- | --- |
| NCT (N-trans-caffeoyltyramine) | syninnova | SL-806 |
| NFT (N-trans-Feruloyltyramine) | syninnova | SL-804 |
| NcisCT (N-Cis-Caffeoyltyramine) | BioBioPha Co., Ltd. | BBP03248 |
| N-Coumaroyldopamine | ARK Pharm | AK122566 |
| p-Coumaroyltyramine | syninnova | SL-808 |
| N-trans-Feruloyloctopamine | Chemfaces | CFS201702 |
| N-p-Coumaroyloctopamine | Chemfaces | CFN97706 |
| Alverine | Sigma | A0424 |
| FTY720 | Sigma | SML0700 |
| DL-Dihydrosphingosine | Sigma | D6783 |
| S1P (Sphingosine 1-phosphate) | Sigma | S9666 |
| B-0027 (DES-1 inhibitor) | Echelon Biosciences | B-0027 |
| GT-11 | Avanti polar lipids | 857395P |
| Ceramide C18 | Avanti polar lipids | 860518P |
| Dihydro-Ceramide C14 | Avanti polar lipids | 860632P |
| Dihydro-Ceramide C16 | Avanti polar lipids | 860634P |
| Dihydro-Ceramide C18 | Avanti polar lipids | 860627P |
| Lalistat 2 | Cayman chemical | 25347 |
| Fenretinide | Cayman chemical | 17688 |
| Rapamycin | Cayman chemical | 13346 |
| RA (all-trans Retinoic Acid) | Cayman chemical | 11017 |
| 4-OXO-RA (4-Oxoretinoic acid) | Sigma | 7558 |
| 5,6-epoxy-RA (all-trans-5,6-epoxy Retinoic acid) | Cayman chemical | 22124 |
| 4-OH-RA (all-trans-4-hydroxy Retinoic acid) | Cayman chemical | 21378 |
| ABT (1-Aminobenzotriazole ) | Cayman chemical | 15252 |
| Talarozole | MedChemExpress | HY-14531 |
